# Positioning uterus transplantation as a ‘more ethical’ alternative to surrogacy: Exploring symmetries between uterus transplantation and surrogacy through analysis of a Swedish government white paper

**DOI:** 10.1111/bioe.12469

**Published:** 2018-07-26

**Authors:** Lisa Guntram, Nicola Jane Williams

**Keywords:** consistency, legislation and policy, reproductive ethics, surrogacy, transplantation ethics, uterus transplantation

## Abstract

Within the ethics and science literature surrounding uterus transplantation (UTx), emphasis is often placed on the extent to which UTx might improve upon, or offer additional benefits when compared to, existing ‘treatment options’ for women with absolute uterine factor infertility, such as adoption and gestational surrogacy. Within this literature UTx is often positioned as superior to surrogacy because it can deliver things that surrogacy cannot (such as the experience of gestation). Yet, in addition to claims that UTx is superior in the aforementioned sense it is also often assumed (either implicitly or explicitly) that UTx is less fraught with ethical difficulties and thus should be considered a less morally problematic option. This article seeks to examine this assumption. Given that much UTx research has been performed in Sweden, a country where surrogacy is *effectively* although not currently *explicitly *forbidden, we do this through an analysis of the arguments underpinning a 2016 Swedish white paper which considered amending existing policy such that altruistic surrogacy arrangements would be permitted. By applying the white paper’s arguments for a restrictive position on altruistic surrogacy to the case of UTx using living altruistic donors we find that such arguments, *if they hold in the case of surrogacy*, apply similarly to UTx. We thus suggest that, for reasons of consistency, a similar stance should be taken towards the moral and legal permissibility of these two practices.

## 1 INTRODUCTION

At present—barring legal, regulatory and financial obstacles to access—women with absolute uterine factor infertility (AUFI)^1^ 1Absolute uterine factor infertility is an umbrella term covering infertility problems in women who either lack a uterus as a result of congenital abnormality or previous hysterectomy or possess a uterus but due to physiological or anatomical abnormalities are unable to conceive or sustain gestation. See Lefkowitz, A., Edwards, M., & Balayla, J. (2012). The Montreal criteria for the ethical feasibility of uterine transplantation. *Transplant International*, *25*, 439. have two options for parenthood: adoption and surrogacy. Recent trials in uterus transplantation (UTx) seek to add a third option. This would allow those desirous of the opportunity, and willing to undertake the risks of at least three major surgeries, to become, not only social and/or genetic parents as they may through adoption and traditional or gestational surrogacy arrangements,^2^ 2Here, traditional surrogacy refers to surrogacy arrangements in which the surrogate is genetically related to the child she gestates (i.e., using her own ova and either donor sperm or the sperm of the intended father) and gestational surrogacy refers to arrangements in which the surrogate is not genetically related to the child she gestates (i.e., where the ova of the intended mother or that of a donor is used). but parents in a social, genetic, *and* gestational sense.^3^ 3Williams, N. J. (2016). Should deceased donation be morally preferred in uterine transplantation trials? *Bioethics*, *30*, 415. Although still experimental, recent trials of the procedure using living donors in Sweden^4^ 4Gustavsson Kubista, M. (2017). *Eight children born after uterus transplants*. Retrieved from October 10, 2017. http://sahlgrenska.gu.se/english/research/news‐events/news‐article/?languageId=100001&contentId=1516702&disableRedirect=true&returnUrl=http%3A%2F%2Fsahlgrenska.gu.se%2Fforskning%2Faktuellt%2Fnyhet%2F%2Fatta‐barn‐fodda‐efter‐livmodertransplantation.cid1516702 and the USA^5^ 5Baylor, Scott & White. (2018). *Second mother who received transplanted uterus gives birth*. Retrieved from April 18, 2018. http://news.bswhealth.com/releases/second‐mother‐who‐received‐transplanted‐uterus‐gives‐birth have resulted in 10 live births. Numerous other small‐scale trials using living and deceased donors are also being performed and planned worldwide^6^ 6For details of additional trials planned worldwide see Williams, *op. cit.* note 3, p. 417; Tan, H.‐K., Tan, B.‐K., Tan, L.‐K., Olofsson, J. I., Dahm‐Kähler, P., & Brännström, M. (2017). Starting a uterus transplantation service: Notes from a small island. *BJOG:* *International Journal of Obstetrics & Gynaecology* Online first. At the first congress of the International Society of Uterus Transplantation, Gothenburg, September 18–19, 2017, it was reported that uterus transplantations also have been performed in Brazil, China, Czech Republic, Germany, India, Serbia and the United States. including, notably, a second 10‐case trial in Sweden.^7^ 7Gustavsson Kubista, *op. cit.* note 4.


As with all novel surgical procedures, discussions have emerged within both the ethics and science literature regarding the extent to which UTx improves upon or offers additional benefits when compared to existing ‘treatment’ options for women with AUFI such as adoption and surrogacy. In this paper, we focus on one specific aspect of such discussions. This concerns the way in which a number of authors—primarily associated with teams conducting scientific research into UTx—have positioned UTx as superior to surrogacy, not only because it can deliver what surrogacy cannot (such as the experience of gestation), but also because it is supposedly a less *morally problematic *alternative.^8^ 8See, for example: Brännström, M., Diaz‐Garcia, C., Hanafy, A., Olausson, M., & Tzakis, A. (2012). Uterus transplantation: Animal research and human possibilities. *Fertility & Sterility*, *97*, 1269–1276; Brännström, M., Johannesson, L., Dahm‐Kähler, P., Enskog, A., Mölne, J., Kvarnström, N., Diaz‐Garcia, C., … Olausson, M. (2014). First clinical uterus transplantation trial: A six‐month report. *Fertility & Sterility*, *101*, 1228–1236; Grynberg, M., Ayoubi, J. M., Bulletti, C., Frydman, R., & Fanchin, R. (2011). Uterine transplantation: A promising surrogate to surrogacy? *Annals of the New York Academy of Sciences*, 1221, 47–53; Olausson, M. (2014). Ethics of uterus transplantation with live donors. *Fertility & Sterility*, *102*, 40–43; Robertson, J. A. (2016). Other women’s wombs: Uterus transplants and gestational surrogacy. *Journal of Law and the Biosciences*, *3*, 71; Tan et al., *op. cit*. note 6; Testa, G., & Johannesson, L. (2017). The ethical challenges of uterus transplantation. *Current Opinion in Organ Transplantation*, *22*(6), 593–597.


Although such authors rarely explicitly state that UTx is less fraught with ethical difficulties than surrogacy, this assumption seems implicit in their work. It is, for example, often claimed that UTx may prove a valuable treatment option for women who live in countries where surrogacy is forbidden by laws or effectively prohibited by restrictive regulations or who, as a result of their personal ethical or religious views, find themselves unable or unwilling to engage in surrogacy arrangements. Underpinning these claims seems to lie the assumption that the thorny ethical and complex regulatory issues raised by surrogacy which have been used, at least in part, to justify restrictive legislation in countries such as Sweden *will not* *engage* *in the case of UTx* *or* *will engage only to a lesser degree*. That is, women should be legally permitted to pursue, and are morally justified in pursuing, UTx in cases and contexts where surrogacy is unavailable to them.

This assumption however warrants closer examination. For, it is not clear that the majority of the ethical arguments against surrogacy fail to engage in the case of UTx, especially where living donors are used. Although we do not aim to make a judgement here on the relative strengths of the ethical arguments against surrogacy—or, indeed, the larger question of whether surrogacy and/or UTx are so problematic that they should be legally prohibited or morally condemned—we do wish to critically assess the view that UTx should be considered *less* morally fraught than surrogacy.^9^ 9For a comparison between living donor uterus transplantation and gestational surrogate motherhood with respect to the principle of equipoise, see Testa, G., Koon, E. C., & Johannesson, L. (2017). Living donor uterus transplant and surrogacy: Ethical analysis according to the principle of equipoise. *American Journal of Transplantation*, *17*, 912–916.


Given that much UTx research has been performed in Sweden—a country in which surrogacy is *effectively* although not currently *explicitly *forbidden through regulations which make both brokering and engaging in surrogacy arrangements impossible—we have chosen to do this through examination of the arguments underpinning a 2016 Swedish white paper.^10^ 10Utredningen om Utökade Möjligheter till Behandling av Ofrivillig Barnlöshet. (2016). *Olika Vägar* *till Föräldraskap*. Swedish Government Official Report (SOU) 2016:11. Stockholm, Sweden: Ministry of Justice. The white paper considers, among other things, whether current legislation and policy should be amended such that altruistic surrogacy arrangements would be permitted in Sweden.^11^ 11It should be noted that subsequent to this paper’s acceptance, in early 2018 the Swedish government decided to retain current policy regarding surrogacy which effectively prohibits its brokering and performance in Sweden. For more details see http://www.regeringen.se/pressmeddelanden/2018/02/modernare‐regler‐om‐assisterad‐befruktning‐och‐foraldraskap/ [Accessed April 23, 2018].


In what follows we examine in turn the major arguments provided in the white paper held to justify this restrictive stance, asking whether, and if so, when and how these might engage in the case of UTx. Such arguments include, but are not limited to, claims that surrogacy may: threaten the autonomy of women; strengthen traditional and problematic views regarding gender roles; exploit women; risk serious harms to children; constitute an unacceptable form of burden‐shifting; and be prohibitively difficult to regulate properly.

We suggest, however, that in the vast majority of cases these arguments will, *if they hold in the case of surrogacy*, similarly apply to Utx, especially in cases where living donors are used. As such, for reasons of consistency, we submit that legislators, policy makers and individuals ought to consider taking a similar stance in relation to the moral and legal permissibility of ‘altruistic’ forms of both surrogacy and UTx using living donors.

## 2 BACKGROUND: SURROGACY IN SWEDEN AND THE 2016 WHITE PAPER

Unlike the majority of EU states, where surrogacy is subject to specific legislation, neither paid nor altruistic surrogacy are clearly regulated in Swedish law. There exist no general or specific provisions either forbidding or permitting surrogacy. However, while not explicitly prohibiting surrogacy arrangements, certain requirements in the regulation of assisted reproduction more generally make surrogacy impossible to access within the Swedish healthcare system. Specifically, legislation and policy require that persons undergoing fertility treatment are married, cohabitating, or in a registered partnership, and that the couple *includes a woman who can carry and give birth to the intended child*.^12^ 12Ministry of Health and Social Affairs. (2006). *Lag (2006:351)* *om* *Genetisk Integritet m.m*. Swedish Code of Statutes 2006:351. Chapter 7:1§, 3§. Since 2016, single women have also been permitted to access IVF and donor insemination treatment provided that the egg is her own and she is able to gestate and give birth to the child.^13^ 13Ministry of Health and Social Affairs. (2016). *Lag* *(2016:18)* *om Ändring i Lagen *(2006:351) *om Genetisk Integritet m.m. *Swedish Code of Statutes 2016:18. As a result of this regulation, same‐sex male couples, lesbian couples in which neither can carry a child, and opposite‐sex couples in which the woman cannot carry a child are unable to access treatment.

Sweden, like many other EU countries such as the U.K. and Germany, has enshrined into legislation the Roman legal principle *Mater semper certa* *est*. This states that, irrespective of whether or not she has a genetic link to the child, the woman who gives birth to a child is that child’s legal mother.^14^ 14Ministry of Justice. (1949). *Föräldrabalk* *(1949:381). *Swedish Code of Statutes 1949:381. Ch. 1: 7§. If the mother of the child is, at the time of delivery, legally married or recently widowed her spouse is presumed by law to be the father of the child.^15^ 15Ibid., Ch. 1:1§. Thus, the process of attaining legal parenthood for intended parents in cases of gestational surrogacy arrangements using both of the intended parents own gametes would, *were it permitted*, be identical to that of ‘närstående (related) adoption’ in Sweden. After the birth, a surrogate and her partner are considered the legal parents of the child. Therefore, in order for both intended parents to attain legal parenthood the intended father’s genetic parenthood must be confirmed, and the surrogate must relinquish her parental rights and responsibilities before the intended mother is permitted to adopt the child.^16^ 16Ministry of Justice. (1949). *Föräldrabalk* *(1949:381). *Swedish Code of Statutes 1949:381. Ch. 4: 3§; Utredningen om Utökade Möjligheter till Behandling av Ofrivillig Barnlöshet, *op. cit. *note 10, p. 34.


As a result of this restrictive legislative stance, there has been much discussion in Sweden in recent years regarding whether or not it would be desirable (and possible) to amend existing legislation and policy surrounding reproduction in order to permit altruistic surrogacy arrangements between those with close emotional ties (such as friends and family members). In 2013, for example, the Swedish National Council on Medical Ethics (SMER) published a report which held that, under certain strict conditions and robust regulations (designed to minimize the harms and risks often associated with surrogacy arrangements for *all parties*) altruistic surrogacy ‘could be an ethically acceptable method of assisted reproduction in Sweden.’^17^ 17Swedish National Council on Reproductive Ethics (SMER). (2013). *Assisted reproduction – Ethical aspects. Summary of a report*. SMER 2013:1. Stockholm, Sweden: SMER, 7. These conditions included the requirements that surrogates and intended parents enjoy a close relationship; that the surrogate has existing children and would not be genetically related to the child; that both parties are screened for suitability, provide valid consent, and have access to support and counselling throughout the process; and that the prospective child is told of her origins and provided with the means, once she reaches majority, to access information regarding the surrogate.^18^ 18Ibid., pp. 6–9.


Yet, despite the conclusions and recommendations of SMER’s report, a white paper commissioned by the Swedish government with the remit of considering ‘different ways to increase the possibilities for involuntarily childless people to become parents’^19^ 19Utredningen om Utökade Möjligheter till Behandling av Ofrivillig Barnlöshet, *op. cit. *note 10, p. 47. and specifically ‘whether to permit surrogacy in Sweden on the basis that it shall, in that case, be altruistic’^20^ 20Ibid. came to the opposite conclusion. For, although the white paper, headed by Eva Wendel Rosberg and published in February 2016, held that permitting surrogacy could provide a number of benefits both within Sweden and internationally,^21^ 21Such benefits included but were not limited to: allowing more involuntarily childless couples to become parents; reducing the numbers of Swedish citizens travelling abroad in order to engage in transnational surrogacy arrangements and thus the legal and moral pitfalls associated with them such as exploitative arrangements, “limping” legal parentage and stateless children; ensuring access to information regarding donor identity on the part of the child once it reaches legal majority; and increasing the freedom of women who wish to act as surrogates for loved ones. For more information see ibid., p. 386. it concluded that ‘the advantages of permitting [altruistic] surrogacy [could not] be held to outweigh the disadvantages.’^22^ 22All translations of the white paper as well as other documents in Swedish were made by Guntram whose first language is Swedish. The disadvantages cited, unsurprisingly, map closely onto those which have, for the past 30 years, been extensively discussed in the ethics literature on surrogacy.^23^ 23The ethics literature surrounding both commercial and altruistic surrogacy is vast but for an insight into some of the key areas of debate surrounding these practices see: Anderson, E. S. (1990). Is women’s labor a commodity? *Philosophy & Public Affairs*, *19*, 71–92; Anleu, S. R. (1992). Surrogacy: For love but not for money? *Gender & Society*, *6*, 30–48; Arneson, R. J. (1992). Commodification and commerical surrogacy. *Philosophy & Public Affairs*, *2*, 132–164; Glover, J. (1989). *Ethics of new reproductive technologies: The* *Glover Report to the European Commission*. DeKalb: Northern Illinois University Press; Oakley, J. (1992). Altruistic surrogacy and informed consent. *Bioethics*, *6*, 269–287; Purdy, L. M. (1989). Surrogate mothering: Exploitation or empowerment? *Bioethics*, *3*, 18–34; Radin, M. J. (1996). *Contested commodities*. Cambridge, MA: Harvard University Press; Robertson, J. A. (1996). *Children of choice: Freedom and the new reproductive technologies*. Princeton, NJ: Princeton University Press; Toledano, S. J. (2016). Sharing the embodied experience of pregnancy: The case of surrogate motherhood. In E. Malmqvist & K. Zeiler (Eds.), *Bodily exchanges, bioethics and border crossing: Perspectives on giving, selling and sharing *bodies (pp. 102–118)*.* Abingdon: Routledge; Wilkinson, S. (2003a). The exploitation argument against commercial surrogacy. *Bioethics*, *17*, 169–187; Wilkinson, S. (2003b). *Bodies for sale: Ethics and exploitation in the human body trade*. London: Routledge. These included claims that surrogacy:

- Threatens the autonomy of women who may experience internal and/or external pressures to enter into surrogacy arrangements and continue with an arrangement once pregnant;
- Strengthens traditional and problematic gender roles and exploits women;
- Risks serious harms to both the children of surrogates and those born through surrogacy arrangements;
- Unacceptably shifts the harms and burdens of reproduction to third parties; and
- Would be prohibitively difficult to regulate appropriately and determine responsibility for costs.^24^ 24Utredningen om Utökade Möjligheter till Behandling av Ofrivillig Barnlöshet, *op. cit. *note 10, pp. 391–445.

The sections that follow critically examine this white paper with the aim of demonstrating that many of the arguments against the introduction of surrogacy in Sweden should also, *if accepted*, challenge the appropriateness of UTx. By highlighting the similarities between these two practices we cast doubt upon the assumption that UTx should be considered a ‘more ethical’ alternative to surrogacy arrangements.

## 3 AUTONOMY, INFORMED CONSENT AND UNDUE PRESSURE

The principle of respect for autonomy arises at several points in the white paper. In discussions of informed consent and risks of pressure it underscores the importance, in liberal societies such as Sweden, of a woman’s right to control her own body and reproductive capacities. Thus, it is noted that women and men should be free ‘to engage in altruistic actions involving the body and its functions, such as donating organs, blood, eggs, and sperm’ despite the fact that such actions may entail ‘pain, inconvenience, and medical risks.’^25^ 25Utredningen om Utökade Möjligheter till Behandling av Ofrivillig Barnlöshet, *op. cit. *note 10, p. 416. Against this backdrop, and given that the white paper finds that the physical and medical inconveniences for surrogate mothers of surrogacy arrangements are not unacceptably high, it holds that ‘psychologically healthy adult women’ ought to be permitted to enter into surrogacy arrangements in cases where it is ‘possible to ensure that the act is voluntary, informed consent can be provided, and there are no other strong reasons against it.’^26^ 26Ibid., p. 417.


However, despite these assertions, the white paper asks two questions. The first concerns whether it is, *in fact*, possible for a surrogate to understand the implications of and thus validly consent to ‘giving away a child that one has carried and given birth to, and if so *when and how *such a consent can be given.’^27^ 27Ibid., p. 392. The second regards when and how to assess whether ‘consent really is voluntary and not, for example, the result of pressure, coercion, or financial incentives.’^28^ 28Ibid., p. 419.


Putting questions of the *possibility of consent* aside, the white paper asserts that, despite existing research providing only a few instances of women having encountered implicit or explicit external pressures to take part in surrogacy arrangements, ‘the risk that a woman may encounter such pressures or experience emotional coercion to act as a surrogate mother cannot be ignored.’^29^ 29Ibid., p. 422. It notes that the risks of external pressures undercutting consent are most likely in cases of altruistic surrogacy where the ‘woman is close to the involuntarily childless individual [or couple]’^30^ 30Ibid. as the surrogate will have witnessed the strength of the individual(s) desire to become (a) parent/s and family and friends often ‘have a strong emotional influence over each other.’^31^ 31Ibid. It also claims, however, that where surrogacy arrangements occur between those without a close emotional relationship there is a greater risk of hidden financial motives. Few specific explanations as to why this would be the case, or how such pressure would be enacted, are provided, as this claim primarily draws on a brief overview of an increase in purportedly altruistic surrogacy arrangements, but which are suspected to be commercial in nature, in Greece.^32^ 32Papaligoura, Z., Papadatou, D., & Bellali, T. (2015). Surrogacy: The experience of Greek commissioning women. *Women and Birth*, *28*(4), e110–e118. This is also one of the few instances where parallels are made to Swedish regulations surrounding live organ donation. Here it notes that the risk of commercialization in live organ donation between individuals without a close relationship ‘has been one of the reasons for the requirement that there should be a close relation between the donor and recipient in live organ donation’^33^ 33Utredningen om Utökade Möjligheter till Behandling av Ofrivillig Barnlöshet, *op. cit. *note 10, p. 423. and recognizes the parallel risks in surrogacy arrangements between those without a close emotional relationship.^34^ 34It should be noted that the Swedish Transplantation Act specifies that “if there are certain reasons such an intervention can be made on another person than those previously stated” which in practice opens up for non‐related live organ donations. See Ministry of Health and Social Affairs. (2006). *Lag* O*m Transplantation m.m. *Swedish Code of Statutes 2006:351, 7§*. *See also, Ministry of Justice. (2015). Utredningen om donations‐ och transplantationsfrågor. *Organdonation. En Livsviktig Verksamhet.* Swedish Government Official Report (SOU) 2015:84. Stockholm, Sweden: Ministry of Justice, p. 464.


Given the risks of both external and internal pressures, the white paper concludes that it would not be possible, in Sweden (or indeed, perhaps anywhere), to create a system which both allows women the freedom to act as surrogates in cases where they can provide consent and protects those whose capacity to consent to entering into surrogacy arrangements has been compromised either internally (by, for example, the prospect of monetary reward) or externally (through coercion or manipulation by friends and family members). The white paper therefore recommends a precautionary approach to risk management in this case, holding that as a result of epistemic limitations it is:

… not possible to—with reasonable measures—create satisfactory guarantees that women are not acting as surrogate mothers because of pressure, because they feel that they have to or because of economic gain. Neither is it possible to create satisfactory guarantees against pressure during the process of the arrangement. This is a strong argument against allowing surrogate motherhood.^35^ 35Utredningen om Utökade Möjligheter till Behandling av Ofrivillig Barnlöshet, *op. cit.* note 10, pp. 425–426.

In what follows, we explore how such arguments might be applied to the case of UTx. Given the precautionary approach to the management of the risk that surrogates may have their capacity to consent compromised advocated by the white paper, it is perhaps unsurprising that UTx would likely not be permitted should it be assessed in a similar manner. Living donor UTx, after all, poses very similar risks to surrogacy. For, unless only altruistic donation by strangers and those already undergoing a hysterectomy is permitted, ‘… donors may be coerced or manipulated into donation by those holding stakes in their donation and … may also feel an internal pressure to donate even in the absence of actively coercive or manipulative acts.’^36^ 36Williams, *op. cit. *note 3, p. 422.


Indeed, just as the white paper asks whether a surrogate can truly understand the implications of giving away the child she has gestated for 9 months^37^ 37Utredningen om Utökade Möjligheter till Behandling av Ofrivillig Barnlöshet, *op. cit. *note 10, p. 392. so too has it been questioned whether a uterus donor can fully understand the ramifications of donating a uterus. For, although total abdominal hysterectomy is considered a routine operation in many nations, uterus donation poses a number of psychological risks similar to those of hysterectomy which include, but are not limited to, feelings of a loss of gender identity and sexual dysfunction.^38^ 38Kisu, I., Banno, K., Mihara, M., Suganuma, N., & Aoki, D. (2013). Current status of uterus transplantation in primates and issues for clinical application. *Fertility & Sterility*, *100*, 288. Indeed, Catsanos et al. note:

A uterus is only expendable if the potential donor is unequivocally certain that she will not now nor in the future desire another pregnancy herself … some of the women who responded to news of UTx had chosen hysterectomy as the solution to a medical problem, thinking they had completed their families, only to find themselves in a new relationship and desirous of having children with their new partner.^39^ 39Catsanos, R., Rogers, W., & Lotz, M. (2013). The ethics of uterus transplantation. *Bioethics*, *27*, 71.

Furthermore, and unlike a routine hysterectomy, but in line with other assisted reproductive technologies, the transfer of a uterus and the subsequent gestation and birth of a child could result in unanticipated relational complexities between the donor, the recipient and the child created. Such complexities could require renegotiations of the meanings accorded to kinship and embodiment especially in contexts where gestational motherhood is accorded greater significance than genetic motherhood.^40^ 40Other assisted reproductive technologies have, after all, been shown to call for new ways to talk about and construe familial bonds and embodiment among the individuals involved in and who result from them. Such work may require emotional as well as relational work, as it involves challenging beliefs and assumptions which have previously been taken for granted. See for example Becker, G. (2000). *The elusive embryo: How women and men approach new reproductive technologies*. Berkeley: University of California Press; Edwards, J., Hirsch, E., Franklin, S., Price, F., & Strathern, M. (1993). *Technologies of procreation: Kinship in the age of assisted conception*. Manchester: Manchester University Press; Franklin, S. B., & McKinnon, S. (Eds.). (2001). *Relative values: Reconfiguring kinship studies*. Durham, NC: Duke University Press; Freeman, T. (2014). *Relatedness in assisted reproduction*. Cambridge: Cambridge University Press; Guntram, L. (2016). Hooked on a feeling? Exploring desires and “solutions” in infertility accounts given by women with “atypical” sex development. *Health*. doi:10.1177/1363459317693403; Provoost, V., Zeiler, K., De Sutter, P., Pennings, G., & Buysse, A. (2017). Constructing and enacting kinship in sister‐to‐sister egg donation families: A multi‐family member interview study. *Sociology of Health &* *Illness*, *39*(6), 847–862; Pashigian, M. J. (2009). The womb, infertility, and the vicissitudes of kin‐relatedness in Vietnam. *Journal of Vietnamese Studies*, *4*, 34–65; Toledano S. J., & Zeiler, K. (2016). Hosting the others’ child? Relational work and embodied responsibility in altruistic surrogate motherhood. *Feminist Theory*, *18*(2), 159–175; Thompson, C. (2005). *Making parents: The ontological choreography of reproductive technologies*. Cambridge, MA: MIT Press. The Swedish UTx team’s reports of possible effects on the relationship between donor and recipient^41^ 41Järvholm, S., Johannesson, L., & Brännström, M. (2015). Psychological aspects in pre‐transplantation assessments of patients prior to entering the first uterus transplantation trial. *Acta Obstetricia et Gynecologica Scandinavica*, *94*, 1037. might not present strong arguments to support claims of UTx having significant and negative consequences for the relationships between participants; their findings do, however, indicate that UTx, in line with other live organ donations, could result in familial and relational conundrums. In light of previous research on such complexities in live kidney and live liver donation,^42^ 42See, for example, Scheper‐Hughes, N. (2007). The tyranny of the gift: Sacrificial violence in living donor transplants. *American Journal of Transplantation*, *7*, 507–511; Zeiler, K., Guntram, L., & Lennerling, A. (2010). Moral tales of parental living kidney donation: A parenthood moral imperative and its relevance for decision making. *Medicine, Health Care & Philosophy*, *13*, 225–236. it could therefore be argued that UTx might result in painful experiences of guilt and responsibility, especially in cases where the donation or transplantation proves unsuccessful or causes significant medical complications.^43^ 43See, for example, Gill, P., & Lowes, L. (2009). The kidney transplant failure experience: A longitudinal case study. *Progress in Transplantation*, *19*, 114–121; Gill, P., & Lowes, L. (2014). Renal transplant failure and disenfranchised grief: Participants’ experiences in the first year post‐graft failure—A qualitative longitudinal study. *International Journal of Nursing Studies*, *51*, 1271–1280; Spiers, J., Smith, J. A, & Drage, M. (2016). A longitudinal interpretative phenomenological analysis of the process of kidney recipients’ resolution of complex ambiguities within relationships with their living donors. *Journal of Health Psychology*, *21*, 2600–2611; Papachristou, C. (2009). Living donor liver transplantation and its effect on the donor‐recipient relationship—A qualitative interview study with donors. *Clinical Transplantation*, *23*, 382–391.


Importantly, in addition to the similar *kinds *of risks and relational complexities posed by living uterus donation and surrogacy, the current state of knowledge regarding the rationales and motivations of uterus donors and the extent to which they have so far experienced pressure or regret is far more limited than in the case of surrogacy. For, despite assertions on the part of the Swedish team that living donors have experienced few psychological sequelae as a result of their donation through both personal communication^44^ 44This was underscored both in our conversations with physicians involved in the Swedish trial during the 2016 symposium held at Lancaster University on the Ethics of Uterus Transplantation and the congress of ISUTx in September 2017 as well as in Guntram’s conversations with the Sahlgrenska team as part of her ongoing research. and media reports,^45^ 45It should, however, be noted that media reports cover recipients who have given birth and not the two who had the transplant removed prior to embryo transfer. See, for example, Björnheden, H., Wiman, L., & Ohlsson, O. (2016, November 15). Ingreppet Gav Tove Barn. *Göteborgs‐Posten*, 6–7; Wikander, F. (2016, June 16). Tack för att Jag Fick din Livmoder! *Amelia*, 29–32; Westman, H. (2015, September 18). Vägen till Vincent. *DI Weekend*, 20–24. there have, as yet, been no published reports of experiences of living uterus donors. Thus, given that the white paper finds the evidence available in the context of surrogacy to be insufficient there is little doubt that the same conclusion should be reached in the case of UTx.

## 4 THE EXPLOITATION AND COMMODIFICATION OF WOMEN

Another key set of concerns running through the white paper regard whether, even where a woman consents to acting as a surrogate, the practice should nevertheless be forbidden in order to avoid both the wrongful exploitation of women and the perpetuation of problematically discriminatory and essentialist views about the ‘proper’ role of women and their bodies.

With respect to the former, for example, although no effort is made within the document to define ‘exploitation’ or what makes a practice *itself *an instance of a practice exploitative in a normative sense,^46^ 46For an excellent discussion of surrogacy and the concept of exploitation see Wertheimer, A. (1992). Two questions about surrogacy and exploitation. *Philosophy & Public Affairs*, *21*, 211–239. concerns are raised regarding whether permitting either or both altruistic and paid surrogacy in Sweden could lead to the exploitation of women both nationally and internationally. Within Sweden, for example, the white paper suggests that forbidding paid surrogacy in Sweden would protect against the exploitation of women with low socioeconomic status who may be unfairly induced into surrogacy by their circumstances. However, despite this, it is also noted that permitting only altruistic surrogacy might be considered exploitative for the opposite reason: that, given the amount of time and effort it takes to gestate a child, surrogates might reasonably expect to be paid for their labour.^47^ 47Utredningen om Utökade Möjligheter till Behandling av Ofrivillig Barnlöshet, *op. cit. *note 10, p. 395.


Similarly, outside Sweden the white paper expresses the concern that permitting altruistic surrogacy within Sweden has the potential to ‘normalize’ surrogacy and lead to a situation in which:

… persons who do not meet the criteria for treatment in Sweden, who do not have access to a surrogate mother, or who do not want to be on the waiting lists that are expected to occur in the health care system, instead choose to hire a commercial surrogate abroad.^48^ 48Ibid., p. 393.

This kind of ‘reproductive tourism’ may well not be considered necessarily exploitative should individuals travel to countries where adequate regulations and safeguards prevent the exploitation of surrogates. However, the concern here is that Swedish couples will travel to countries where surrogacy is comparatively cheap or regulated less stringently, which is more likely to lead to the exploitation of poor women. Thus, as demonstrated, concerns expressed within the white paper closely map on to those which have been discussed extensively in the ethics literature surrounding exploitation and surrogacy in both developed and developing nations.^49^ 49Notable examples of this literature include: Ber, R. (2000). Ethical issues in gestational surrogacy. *Theoretical Medicine and Bioethics*, *21*, 153–169; Anderson, E. S. (1990). Is women’s labour a commodity? *Philosophy & Public Affairs*, *19*, 71–92; Wertheimer, *op. cit.* note 46, pp. 211–239; Wilkinson (2003b), *op. cit. *note 23, Ch. 8; Wilkinson (2003a), *op. cit. *note 23, pp. 125–145; Field, M. (1989). Surrogate motherhood: The legal and human issues (expanded edition). Cambridge, MA: Harvard University Press, Ch. 2; Baylis, F. (2014). Transnational commercial contract pregnancy in India. In F. Baylis & C. Macleod (Eds.), *Family making: Contemporary ethical challenges *(pp. 265–286). Oxford: Oxford University Press.


In terms of discrimination the white paper suggests that, even in the absence of concerns regarding the exploitation of surrogates, there may still be good reasons to refrain from permitting surrogacy as part of the wider project of securing equality for women by rejecting traditionalist, essentialist and patriarchal views regarding gender and the role of women as ‘givers/providers’^50^ 50Utredningen om Utökade Möjligheter till Behandling av Ofrivillig Barnlöshet, *op. cit.* note 10, p. 395. who are essentially or, most importantly, gestators and mothers. That permitting surrogacy risks perpetuating such views, however, is not held to constitute sufficient reason to forbid surrogacy in Sweden as it is noted that just as some authors view surrogacy to be necessarily damaging to women, others suggest it has the potential to prove emancipatory, strengthening the woman’s autonomy to control her own body and its processes.^51^ 51Ibid., p. 435.


A linked concern, raised in the white paper with respect to the Swedish context, regards the moral acceptability of ‘burden‐shifting’ in reproduction and whether surrogacy is appropriate as the risks and costs associated with reproduction are shifted from the intended parents on to a third party. When read at face value this is an odd argument, given that the majority of paid labour constitutes burden‐shifting. However, while shifting the burdens of reproduction on to third parties is not necessarily morally problematic for those who lack the ability to gestate and birth their future children, permitting surrogacy in such cases could lead to an increased acceptance of ‘convenience’ surrogacy in Sweden which, although posing few problems for the wealthy, could lead to the further marginalization of less advantaged women.^52^ 52The view that shifting the burdens of reproduction may be considered more objectionable in cases of convenience (i.e., where it is not motivated by medical reasons such as an inability to conceive or sustain gestation) has also been expressed in government reports of other nations regarding surrogacy such as that of the U.K.’s Warnock report: Warnock, M. (1984). *Report of the Committee of Inquiry into Human Fertilisation and Embryology*. London: Her Majesty’s Stationery Office. S.8.17.


As in the previous section of this article, however, it seems that very similar concerns regarding exploitation and the perpetuation of discriminatory and problematic views regarding women and their bodies can be, and indeed, have been, raised in the context of UTx.

Just as concerns are expressed that permitting ‘altruistic’ surrogacy in Sweden may encourage women unable to find a surrogate in Sweden to go abroad and engage in paid surrogacy, so too have concerns been raised regarding the potential for the creation of a ‘black market’ in uteri similar to that seen in kidneys.^53^ 53For a general account of such worries in the context of UTx see Dickens, B. M. (2016). Legal and ethical issues of uterus transplantation. *International Journal of Gynecology & Obstetrics*, *133*, 126–127. For, it is possible that women who fail to meet the selection criteria for UTx in their ‘home’ countries, lack the necessary financial resources to pay for their own and their donor’s medical expenses in their home country, and/or are unable to find friends and family members willing to donate may look further afield, seeking to purchase uteri from women who find themselves in such precarious economic positions that they are willing to sell their uteri. In a paper regarding UTx in the Middle East, Altawil and Arawi express this concern, noting that just as some Syrian refugee families have:

resorted to selling organs to make ends meet … it is very conceivable that impoverished families, especially refugees that have found themselves in a dire financial situation, may resort to selling their or their daughters’ uteri in order to be able to survive.^54^ 54Altawil, Z., & Arawi, T. (2016). Uterine transplantation: Ethical considerations within Middle Eastern perspectives. *Developing World Bioethics*, *16*, 91–97.

They thus ask whether it would be morally justifiable to introduce uterine UTx given that it ‘has the potential to become another vehicle for human expoitation.’^55^ 55Ibid.


More generally, discussions have also emerged within the ethics literature regarding whether both permitting (and/or providing public support for) UTx may reinforce the damaging sexist and pronatalist beliefs regarding the importance of gestation and genetic relatedness for both womanhood and parenthood. Thus, the concern here is that a government’s acceptance (and provision) of UTx may collude with and perpetuate the erroneous and discriminatory beliefs which exacerbate the pain and suffering experienced by many infertile women. As a result they may also be more likely to discount other options for parenthood, such as adoption, in favour of undergoing risky and expensive procedures and interventions aimed at replicating more traditional methods of founding a family.^56^ 56For an insight into this debate, see Wilkinson, S., & Williams, N. J. (2016). Should uterus transplants be publicly funded? *Journal of Medical Ethics*, *42*(9), 559–565; Lotz, M. (2016). Commentary on Nicola Williams and Stephen Wilkinson “Should uterus transplants be publicly funded?” *Journal of Medical Ethics*, *42*, 570–571; Wilkinson, S., & Williams, N. J. (2016). Public funding, social change and uterus transplants: A response to commentaries. *Journal of Medical Ethics*, *42*, 572–573. This again could be especially worrying in countries where ‘social respect for women is still very much connected to their ability to give birth to a child.’^57^ 57Altawil & Arawi, *op. cit. *note 54, p. 97.


When it comes to concerns regarding burden‐shifting we acknowledge that it would be nonsensical to argue that those desirous of uterus transplants seek to shift the burdens of gestation and birth on to others. That UTx avoids raising such concerns, has been noted by scholars such as Grynberg et al.^58^ 58Grynberg et al., *op. cit.* note 8, p. 47. and Olausson.^59^ 59Olausson, *op. cit.* note 8, p. 40. Indeed, in a recent paper examining the possibility of a duty to choose UTx over surrogacy (once UTx becomes safe and effective) John Robertson rather startlingly notes:

The discomfort with surrogacy for convenience shares a kindred ethical root as the argument for uterus transplant instead of surrogacy. Market power allows a woman to hire the surrogate even when she could physically gestate herself. If we are troubled by convenience cases, then we should be troubled by women who reject safe and effective transplants and hire [other] women to gestate [their children].^60^ 60Robertson, *op. cit. *note 8, p. 77.

Yet whilst living donor UTx does allow the intended mother to shoulder the physical burdens and risks associated with gestation and childbirth, and thus reduces the risks undertaken by third parties, living uterus donation is not risk free. Deceased uterus donation, if shown to be successful, would reduce such risks even further and thus might also be considered preferable if we are concerned to ensure that the burdens of reproduction are carried, insofar as is possible, by those who stand to reap its benefits. On a slightly different note, UTx using living donors may well also shift *feelings of responsibility *for the burdens and harms resulting from uterine factor infertility on to the female family members and friends of those who seek UTx.^61^ 61This concern is closely tied to those discussed in the previous section of this article regarding undue pressure and coercion.


## 5 THE WELFARE OF THE CHILD

In Sweden^62^ 62Human Fertilisation and Embryology Act 1990 s. 13 (5). it is held that in matters affecting the interests of children, both future and existing, their welfare must be taken into account, and thus in the context of assisted reproduction, practices and policies which benefit prospective parents *but* pose significant risks of physical and/or psychological harm to children are prohibited. Thus, it is perhaps unsurprising that another concern discussed at length in the white paper is whether surrogacy is compatible with the welfare of both children born through surrogacy arrangements and surrogates’ existing children.

The white paper suggests that although permitting surrogacy in Sweden has the potential to reduce some of the harms to children created which have arisen as a result of Swedish parents engaging in transnational surrogacy arrangements—such as ‘limping legal parentage,’^63^ 63This term refers to situations in which after a surrogacy arrangement legal parentage is granted in one state but not in another, namely, the state in which the intended parents of the surrogacy‐conceived child are resident. statelessness, and an inability to access information regarding the identity of their surrogates and (where applicable) gamete donors^64^ 64Utredningen om Utökade Möjligheter till Behandling av Ofrivillig Barnlöshet, *op. cit.* note 10, p. 390.—it also risks a number of serious harms to children which ‘speak strongly’ against permitting surrogacy in Sweden.^65^ 65Ibid., p. 415. In terms of surrogate‐conceived children, the risks explored and highlighted include the possible harms and familial instability that the child created may face should the surrogacy arrangement go awry; and the possible negative impact that being born through surrogacy may have on identity formation, attachment during the early years, and family relationships during the later years of childhood.^66^ 66Ibid., pp. 410–414. Concerns, however, are also raised regarding the welfare of surrogates’ existing children as it is suggested that such children may, depending on their ages, experience fears that they too will be ‘given away’ or feel jealousy and anger towards the prospective child during pregnancy, or worry for the child after his/her birth.^67^ 67Ibid., p. 414.


The white paper does acknowledge however that, just as in the case of discussions of informed consent and risks of pressure and coercion, there is little evidence available regarding the outcomes of children born through surrogacy or the children of surrogates. It recognizes that the few studies undertaken regarding these outcomes suggest there is little reason to assume they will fare any worse in terms of familial relationships and psychological welfare than other children.^68^ 68Ibid., p. 412. However, despite this, the white paper notes a number of problems regarding the data available—such as that the majority of the available studies on outcomes have been conducted by one U.K. research group (The Centre for Family Research at Cambridge University); no long‐term studies have occurred; and there is a notable lack of studies focusing on the outcomes for children in cases of conflict between the surrogate and intended parents.^69^ 69Ibid., p. 415. As such, the white paper again suggests a ‘precautionary approach’—placing the burden of proof on those who would claim that surrogacy is not harmful—and that uncertainties regarding the effects of surrogacy on child welfare, ‘strongly speak against permitting surrogacy’^70^ 70Ibid., p. 415. in Sweden.

Were such an approach translated into the context of UTx, a similar conclusion would almost certainly be reached. This is so for two reasons. First, whilst surrogacy is an established practice, UTx is not and thus there is even less evidence available to deny or support the claim that it is compatible with the best interests of children. Thus, given the precautionary approach advocated by the authors in the case of surrogacy, a lack of evidence supporting the claim that UTx is not likely to cause significant harm to children should, for reasons of consistency, be held to ‘strongly speak against permitting’ UTx in Sweden too. Second, is that although there is little empirical evidence at this time, it does not seem unreasonable to suggest that being gestated in a donated uterus is likely to prove more risky than (or, at the very least, as risky as) being born through surrogacy in terms of negative effects on child welfare.^71^ 71Daar, J., & Klipstein, S. (2016). Refocusing the ethical choices in womb transplantation. *Journal of Law and the Biosciences*, *3*, 388. For, whilst the risks to the physical health of children born through surrogacy are similar to those of reproduction more generally, concerns have been forwarded suggesting that children born through UTx are more likely to experience physical harms during pregnancy which could have grave effects upon their future welfare. Daar and Klipstein summarize these risks as follows:

The medical literature suggests that gestating a foetus in a transplanted uterus poses several risks to the developing child, including (i) the potential for compromised uterine blood flow and its effect on the developing fetus, (ii) concomitant maternal renal abnormalities associated with some of the conditions that result in an absent or malformed uterus which may increase the risk of preeclampsia and hypertension, and (iii) the potential fetal teratogenic effects of exposure to immunosuppressants.^72^ 72Ibid., p. 384. In order to retain balance it should be noted that a somewhat different (and less negative) take on the physical risks for children born through UTx is presented in the following paper: Testa et al., *op. cit. *note 9, pp. 912–916.

Given this, *if *a concern to act in the best interests of children is sufficient to justify prohibiting surrogacy in Sweden, the same would seem to go for UTx: i.e., it too should be prohibited.^73^ 73It should be noted, however, that although concerns regarding child welfare *can* be forwarded against both UTx and surrogacy, questions remain regarding whether the suggestion that one ought to prohibit either surrogacy or UTx *because* children born as a result of surrogacy arrangements or UTx *may* experience lower levels of welfare than those born in a more traditional manner, is philosophically defensible. For, given that it can be relatively safely assumed that once born children created through both surrogacy and UTx are likely to have lives that are worth living it is more than a little perverse to suggest that their welfare ought to be protected by preventing their very existence.


## 6 REGULATORY DIFFICULTIES

Another concern identified in the white paper regards the possibility of designing an appropriate regulatory framework for surrogacy in Sweden given tensions between the rights and interests of surrogates, intended parents and the children created through surrogacy; and difficulties in determining and enforcing responsibility for the costs surrogacy arrangements could impose on the Swedish health system, employers and social services.

Regarding the former, two possible tensions between the interests of such parties are discussed:

1. The surrogate’s interest in controlling her own body and the interests of the intended parents and the potential child in ensuring a healthy birth.
2. The right of the surrogate to choose to take care of the child she has gestated and given birth to and the right of the intended parents to take care of the child who is genetically related to them and who exists only because of their actions.

It is noted, for example—through reference to one case in which a surrogate mother, and the intended father, opposed the intended mother’s application to adopt the child^74^ 74In this particular case the surrogate mother was the sister of the intended and genetic father and the intended mother was the genetic mother of the child. See Högsta Domstolen NJA (2006), p. 505.—that although ensuring that surrogacy arrangements are not enforceable either during pregnancy or for a number of weeks post‐birth protects the interests and rights of the surrogate, this results in uncertainty for intended parents and may cause them significant stress and worry. Similarly, and more dramatically, it is suggested that in rare circumstances some surrogates would abuse a right to withdraw their consent by demanding compensation or favours from the intended parents, and thus taking advantage of their precarious situation in order to follow through with the arrangement.^75^ 75Utredningen om Utökade Möjligheter till Behandling av Ofrivillig Barnlöshet, *op. cit. *note 10, p. 437.


Duplicitous surrogates however, are not the only concern, as the white paper notes that intended parents may also, in rare circumstances, change their minds partway through the arrangement, and refuse to take the child once born, such as where it is discovered that the fetus has a congenital abnormality liable to result in disability.^76^ 76Ibid., p. 440. This forces the surrogate into a difficult situation where she must decide whether to abort the pregnancy or to keep or put the child up for adoption once born. As a result of these concerns, the authors assert that it is difficult, if not impossible, to create a regulatory framework for surrogacy that balances the interests of the different parties should one of them change their mind. Indeed, irrespective of the chosen solution, the white paper concludes that situations may occur in which the child fares badly. Thus, the presence of such difficulties is held to constitute ‘a strong reason against allowing surrogate motherhood in Sweden.’^77^ 77Ibid., p. 440.


The white paper also discusses regulatory issues relating to financial questions such as: who should be held financially responsible for meeting the healthcare and other costs of surrogacy, especially in the case of complications and whether, when, and if so, how much, compensation should be provided to surrogates. In the Swedish health insurance system, the employer of the pregnant woman covers part of the cost if a pregnant woman needs to reduce her work hours or needs to be on sick leave as a result of the pregnancy. In light of this the white paper asks whether it, for example, is reasonable that these costs should be covered by the surrogate mother’s health insurance and by her employer. In addition, pregnancy generates other expenses connected to treatment, pregnancy and delivery. Again, the white paper asks, who is to cover such costs? In response to this, two main options are discussed: the state and intended parents. While the white paper asserts that a model in which the state covers income losses not covered by the social security system or by the surrogate’s own insurance at least in principle would be the least dubious option, it is concluded that it would—in light of the public costs that will be generated—still be difficult to find a model that is both acceptable in principle and reasonable.^78^ 78It should be noted however, that the white paper does not elaborate on reasons behind the anticipated difficulties. See Utredningen om Utökade Möjligheter till Behandling av Ofrivillig Barnlöshet, *op. cit. *note 10, p. 444.


What happens when similar arguments are applied to UTx? We recognize that the parallels with the case of UTx are not necessarily straightforward, at least not with respect to the first of the two regulatory questions. Far fewer concerns are anticipated regarding how to balance the interests and rights of the uterus donors and recipients. In terms of respect for autonomy, for example, there is little reason to assume that a uterus donor would not, like all other organ donors in Sweden, have the right to withdraw consent at any time prior to the retrieval and transplantation of the uterus^79^ 79Ministry of Health and Social Affairs. (2014). *Patientlag*. Swedish Code of Statutes 2014:821. Ch. 4, 2§. and that, once transplanted, the recipient would, given her right to control her own body, possess the right to request the removal of the uterus and/or to control her pregnancy should the transplant and subsequent embryo transfers prove successful. There could, of course, be scenarios in which a uterus donor attempts to exert control over or influence the decisions of the recipient; or *expects *access to, and a relationship with, the child once born as has been reported in surrogacy arrangements. However, it seems unlikely that a donor would, for example, seek the removal/return of the donated uterus.^80^ 80It should be noted that neither the Swedish regulation of organ transplantation nor any other related act explicitly states that a live organ donor can have the organ returned upon demand or that the organ donor cannot have the organ returned upon demand. See Ministry of Health and Social Affairs. (1995). *Lag* O*m Transplantation m.m.* Swedish Code of Statutes 1995:831, p. 351. Indeed, since the *mater est* principle would apply in the case of UTx, it does not introduce the same legal uncertainties in terms of assigning parenthood. Thus, in this respect, UTx does seem to pose fewer regulatory complexities than surrogacy.

With regard to the second regulatory difficulty, however, it does seem that UTx raises similar issues in certain respects. In terms of direct financial compensation for donors, UTx does seem to raise less complicated questions as the recovery time associated with hysterectomies is (provided the retrieval and recovery are routine) far less disruptive to the life of the donor than surrogacy, especially where post‐menopausal donors are used. However, whilst this is so, UTx using living donors is liable to pose regulatory challenges when it comes to determining who should be responsible for meeting the costs of the retrieval surgery, possible complications, and recovery period. Given this, and just as the white paper on surrogacy raises the question of who is responsible for the surrogate’s potential loss of income and other costs associated to the arrangement, the question of *who *should be responsible for the costs of the retrieval surgery would be equally prominent in the case of UTx.

On the one hand, it would perhaps not be unreasonable to expect that live uterus donors would fall within the same model of compensation as other live organ donors or within the same model as donors of reproductive materials, where the state compensates the donor for income loss and similar associated costs. If so, UTx would not imply any specific regulatory challenges in this respect. On the other hand, however, these questions tie into the broader discussions of whether infertility treatments should be publicly funded and, more specifically, into the complexities of priority setting in the context of UTx, which would need to be taken into careful consideration in the case of UTx being introduced in public healthcare.^81^ 81Wilkinson & Williams, *op. cit.* note 56, pp. 559–565.


In light of the above, we find that in comparison to surrogacy it seems reasonable to position UTx as likely to pose fewer, and less complicated, regulatory questions than surrogacy. However, while it might be a less complicated alternative this does not necessarily make UTx a more *ethical *alternative. Furthermore, in teasing out the regulatory similarities and differences between surrogacy and UTx it is also possible to raise the more general question as to whether, and if so to what extent, anticipated regulatory difficulties should function as arguments against the introduction of novel healthcare practices.

## 7 CONCLUSION

Throughout this paper we have explored whether it is reasonable to claim that, for involuntarily childless women with AUFI, UTx using living donors is a morally superior option to altruistic surrogacy. This question was chosen because, within the ethics and science literature surrounding UTx, it is often implicitly assumed that UTx is preferable to surrogacy, not only because it offers what surrogacy cannot (gestation), but because it is an option that is less fraught with ethical and regulatory difficulties and quandaries than surrogacy and should thus be preferred for *moral reasons*.

As it would have been impossible within this article to examine *all *possible arguments against surrogacy, and in order to situate our discussions and conclusions in a real‐life policy context, we chose to do this through examination of the key arguments forwarded in a recent Swedish white paper which considered whether existing legislation and policy should be amended in order to permit altruistic surrogacy. However, as has been shown above, the assumption that UTx is morally superior to surrogacy does not survive close scrutiny (at least in cases where the arguments considered in the white paper are forwarded against surrogacy). For, where such arguments hold, they also seem to hold straightforwardly in the context of UTx using living donors.^82^ 82While our analysis shows that UTx using living donors should *not* automatically be considered any less morally problematic than surrogacy, it does seem that a case can be made in support of a moral preference for UTx over surrogacy where uteri for transplantation are obtained from deceased donors. After all, whilst the use of deceased donors in UTx is not exactly morally unproblematic, it does seem that many of the ethical arguments which may be forwarded against surrogacy and living donor UTx seem to engage only to a lesser degree in the case of deceased donor UTx. This may not be the case for all concerns, such as those concerning the welfare of the child. However, provided one subscribes to the Epicurean belief that one’s interests cannot survive one’s death, concerns regarding respect for autonomy, informed consent, undue pressure, exploitation and commodification, although still warranted in the case of deceased donors, seem far more pressing in both surrogacy and living donor UTx as only in living donor UTx and surrogacy may the donor/gestator experience harm as a result.


That these two practices raise similar issues is important for reasons of both fairness *and* coherence. When it comes to questions of the laws and principles that govern our behaviours, consisistency is a virtue and like cases should be treated alike. Thus, given that arguments often forwarded against surrogacy seem, for the most part, to apply similarly to Utx, our prescriptions regarding UTx ought to be informed by the position taken regarding surrogacy. They should differ in content only in so far as relevant differences—in, for example, the kinds of ethical problems they raise, their gravity when considered individually or taken together, and the likelihood of their occurrence—can be identified.

No two practices (or indeed, instances of the same practice) are ever identical but we suggest that given the similarities that can be identified there is little reason to assume that UTx is any less morally problematic all things considered. With this in mind, scholars would be wise to refrain from unreflectively suggesting that UTx is less morally problematic than surrogacy without explaining *exactly why they have come to this conclusion *and policy makers in countries such as Sweden, which take a hard line against altrusitic surrogacy, must consider the possibility that forbidding all forms of surrogacy may well be inconsistent with permitting UTx using living donors.

